# 维奈克拉联合多药化疗治疗复发难治早期前体T淋巴细胞白血病15例疗效及安全性分析

**DOI:** 10.3760/cma.j.issn.0253-2727.2023.08.006

**Published:** 2023-08

**Authors:** 金玉 孔, 李红 宗, 妍 浦, 吟 刘, 欣 孔, 梦云 郦, 剑 张, 宝全 宋, 胜利 薛, 晓文 唐, 惠英 仇, 德沛 吴

**Affiliations:** 苏州大学附属第一医院血液内科，江苏省血液研究所，国家血液系统疾病临床医学研究中心，苏州 215006 The First Affiliated Hospital of Soochow University, Jiangsu Institute of Hematology, National Clinical Research Center for Hematologic Diseases, Suzhou 215006, China

**Keywords:** 维奈克拉, 白血病，早期前体T淋巴细胞，急性, 难治, 复发, 抗肿瘤联合化疗方案, Venetoclax, Leukemia, early T-cell precursor, acute, Relapsed, Refractory, Combined chemotherapy

## Abstract

**目的:**

探讨维奈克拉（Venetoclax，Ven）联合多药化疗治疗复发难治早期前体T淋巴细胞白血病（R/R ETP-ALL）患者的疗效及安全性。

**方法:**

回顾性分析2018年12月至2022年2月在苏州大学附属第一医院住院治疗的15例R/R ETP-ALL患者的临床资料。再诱导治疗以Ven为基础联合多药化疗，其中8例联合去甲基化药物，4例同时联合去甲基化药物和HAAG方案，2例同时联合去甲基化药物和CAG预激方案，1例联合克拉屈滨。Ven用法为100 mg第1天，200 mg第2天，400 mg第3～28天，口服；联合唑类抗真菌药物时减量至100 mg/d。

**结果:**

15例R/R ETP-ALL患者中，男10例，女5例，中位年龄35（12～42）岁。难治4例，复发11例。用药第21天疗效：完全缓解（CR）率为60.0％（9/15），CR伴血液学不完全恢复（CRi）率为6.7％（1/15），总有效率（ORR）为66.7％。所有患者12个月总生存（OS）率为60.0％，中位OS时间为17.7个月；全部CR患者12个月无病生存（DFS）率为60.0％，中位DFS时间未达到。14例（93.3％）患者发生了3级及以上血液学不良反应，所有患者经治疗后造血功能恢复且无致死性大出血发生；无患者出现神经系统不良反应及肿瘤溶解综合征，同时无3级及以上脏器不良反应发生。

**结论:**

Ven联合多药化疗治疗R/R ETP-ALL疗效值得肯定，治疗相关不良反应可耐受。

## Abstract (en)

**Objective:**

To explore the efficacy and safety of Venetoclax combined with multidrug chemotherapy in patients with relapsed or refractory early T-cell precursor acute lymphoblastic leukemia（R/R ETP-ALL）.

**Methods:**

This study retrospectively analyzed 15 patients with R/R ETP-ALL who received Venetoclax combined with multidrug chemotherapy from December 2018 to February 2022. Among them, eight cases were combined with demethylated drugs, four cases were combined with demethylated drugs and HAAG chemotherapy regimen, two cases were combined with demethylated drugs and CAG regimen, and one case was combined with Cladribine. Specific usage and dosage of Venetoclax: 100 mg on day 1, 200 mg on day 2, 400 mg on day 3–28, orally; when combined with azole antifungal drugs, dosage was reduced to 100 mg/d.

**Results:**

Fifteen patients（10 males and 5 females）with R/R ETP-ALL were treated with Venetoclax and multidrug chemotherapy with a median age of 35（12–42）years old. Of 4 refractory and 11 relapsed patients, the efficacy was evaluated on the 21th day following combined chemotherapy: the overall response rate, the complete response（CR）rate, and the CR with incomplete hematological recovery（CRi）rate were 67.7％（10/15）, 60.0％（9/15）, and 6.7％（1/15）, respectively. For the overall study population, the 12-month overall survival（OS）rate was 60.0％, and the median OS was 17.7 months. The disease-free survival（DFS）rate of all CR patients at 12 months was 60.0％, and the median DFS did not reach. About 14 patients had Ⅲ–Ⅳ hematological toxicity, but these adverse reactions were all controllable. No adverse reaction in the nervous system and tumor lysis syndrome occurred in this study, and no adverse reaction of organs above grade Ⅲ occurred.

**Conclusion:**

Venetoclax combined with multidrug chemotherapy may be a safe and promising treatment option for patients with R/R ETP-ALL.

早期前体T淋巴细胞白血病（ETP-ALL）是急性T淋巴细胞白血病（T-ALL）的一种特殊类型，发病率低，约占T-ALL的17％[1]，在分子免疫表型、细胞遗传学及预后等方面具有高度的异质性，具有对常规化疗耐药、复发风险高及预后差的特点[2]–[3]。异基因造血干细胞移植（allo-HSCT）已被证实可提高ETP-ALL患者的生存率[4]–[5]，对于复发难治（R/R）ETP-ALL患者，行allo-HSCT前再次实现完全缓解（CR）至关重要。然而，复发及耐药后的最佳再诱导治疗方案尚无定论。BCL-2抑制剂维奈克拉（Ven）是一种小分子抑制剂，直接与BCL-2蛋白结合，置换促凋亡蛋白并恢复凋亡过程，ETP-ALL高度依赖BCL-2，在体外实验和体内治疗中对Ven均敏感。现将本中心应用Ven联合去甲基化药物及预激方案治疗的15例R/R ETP-ALL患者的疗效及安全性报道如下。

## 病例与方法

1. 病例：以2018年12月至2022年2月在苏州大学附属第一医院住院治疗的15例R/R ETP-ALL患者为研究对象，回顾性分析患者的临床资料，诊断符合《中国成人急性淋巴细胞白血病诊断与治疗指南（2021年版）》[6]，所有患者根据骨髓细胞形态学、免疫表型分析、细胞遗传学、分子生物学（MICM）进行诊断分型并确诊。

2. 治疗方法：再诱导治疗以Ven为基础联合多药化疗。Ven标准方案：Ven 100 mg第1天，200 mg第2天，400 mg第3～28天，口服。药物用量及疗程根据不良反应及一般状况调整，1例患者因中性粒细胞减少28 d内均采用100 mg/d，因出现粒细胞缺乏（粒缺）需要伏立康唑预防真菌感染的患者，Ven减至100 mg/d，重度粒缺（<0.2×10^9^/L）合并重度感染患者暂停用药。联合用药方案包括Ven+阿扎胞苷（AZA）、Ven+地西他滨（DAC）、Ven+克拉屈滨（Cladribine）、Ven+DAC+HAAG或Ven+DAC+CAG方案。联合用药方案中除Ven外其他药物的用法：Ven+AZA方案：AZA 75 mg/m^2^第1～7天，静脉滴注。Ven+DAC方案：DAC 20 mg/m^2^第1～5天，静脉滴注。Ven+Cladribine：Cladribine 10 mg第1～3天，静脉注射。Ven+DAC+HAAG方案：DAC 20 mg/m^2^第1～5天，静脉滴注；高三尖杉酯碱1 mg第3～16天，静脉滴注；阿糖胞苷10 mg/m^2^第3～16天，皮下注射；阿柔比星10 mg第3～10天，静脉滴注；G-CSF根据血象调整，皮下注射。Ven+DAC+CAG方案：DAC 20 mg/m^2^第1～5天，静脉滴注；阿克拉霉素20 mg第3～6天，静脉滴注；阿糖胞苷10 mg/m^2^第3～14天，皮下注射；G-CSF根据血象调整，皮下注射。骨髓抑制期HGB<60 g/L或出现明显症状时输注悬浮红细胞，PLT<20×10^9^/L或出现出血倾向时输注血小板。

3. 疗效和不良反应评估：疗效分为①CR：外周血无原始细胞，无髓外白血病；骨髓三系造血恢复，原始细胞<5％；外周血ANC>1.0×10^9^/L且PLT>100×10^9^/L；4周内无复发。②CR伴血液学不完全恢复（CRi）：外周血ANC≤1.0×10^9^/L和（或）PLT≤100×10^9^/L，其他满足CR的标准。③未缓解（NR）：未达到CR和CRi。总有效率（ORR）指患者在治疗后达到CR和CRi的比例。疾病复发指已取得CR的患者外周血或骨髓又出现原始细胞（比例>5％），或出现髓外疾病。难治性疾病定义为诱导治疗结束（一般指4周方案或Hyper-CVAD方案）未能取得CR/CRi[6]。总生存（OS）时间为开始应用Ven至患者死亡或末次随访的时间。无病生存（DFS）时间为获得CR的患者，从获得CR之日起至复发或发生死亡的时间。中性粒细胞减少定义为外周血ANC<2.0×10^9^/L，粒缺定义为外周血ANC<0.5×10^9^/L，血小板减少定义为外周血PLT<100×10^9^/L。造血功能恢复定义为ANC连续3 d≥0.5×10^9^/L，PLT连续3 d≥20×10^9^/L且脱离输注。药物不良反应根据美国国家癌症研究所常见不良反应事件评价标准（CTCAE）5.0版进行报告和分级。

4. 随访：采用电话、查阅患者住院病历及门诊病历的方式随访，随访截止日期为2022年12月30日，中位随访时间为25.0（14.5～35.5）个月，所有患者均未失访。

5. 统计学处理：采用SPSS 26.0软件分析数据，计数资料以例数表示；生存分析采用Kaplan-Meier法，差异性检验采用Log-rank法，检验水准*α*＝0.05，*P*<0.05为差异有统计学意义。

## 结果

1. 一般资料：15例R/R ETP-ALL患者中，男10例，女5例，中位年龄35（12～42）岁。难治4例，复发11例，其中化疗后复发5例，移植后复发6例。11例复发患者既往化疗疗程中位数为2（2～3）个；4例难治患者既往化疗疗程中位数为4（2～10）个。2例伴髓外复发。化疗前血常规（中位数）：WBC 16.6（1.7～498.6）×10^9^/L，HGB 88（55～188）g/L，PLT 85（9～876）×10^9^/L。中位骨髓原始细胞比例71％（17％～99％）。复杂核型3例，异常非复杂核型2例。15例患者二代测序共检出34种共54个基因突变（表1）。

**表1:** 15例复发难治早期前体T淋巴细胞白血病患者的临床资料

例号	诊断	年龄（岁）	性别	初诊WBC（×10^9^/L）	初诊骨髓原始细胞（%）	染色体	基因突变
1	难治	41	女	1.7	71	复杂核型^a^	NOTCH1、BRCA1、PTPN11
2	难治	37	女	2.5	63	46，XX	TP53、FAT1、SUZ12
3	难治	35	女	18.1	74	复杂核型^b^	TP53、ASXL1、CBL、FLT3-ITD、FLT3-KTD、NOTCH1、ETV6、PHF6
4	难治	26	男	15.2	60	46，XY	FBXW7、GATA3、JAK2、JAK3、NF1、KRAS、PALB2
5	化疗后复发	42	女	3.2	56	46，XX,i(9)(q10)[8]/46,XX[2]	NOTCHI、JAK3、IKZF1、JAK2、JAK1、ASXL1
6	化疗后复发	35	男	24.1	88	47，XY，+7[7]/46，XY[3]	NOTCH1、RUNX1、STAT5B、WT1
7	化疗后复发	23	男	20.5	20	不详	不详
8	化疗后复发	33	男	120.0	99	46，XY	NOTCH1、SIL-TAL1
9	化疗后复发	29	男	498.6	61	46，XY	PTEN、SIL-TAL1
10	移植后复发	12	男	2.2	17	46，XY	阴性
11	移植后复发	27	男	82.3	90	46，XY	阴性
12	移植后复发	38	男	3.3	84	46，XY	E2H2、NOTCH1、ETV6、NRAS、JAK3
13	移植后复发	37	女	2.2	64	46，XX	G13D、ASXL2、SETD2、PDGFRA
14	移植后复发	35	男	26.1	78	复杂核型^c^	NOTCH1、DNMT3A、RUNX1、PHF6、IL7R、SUZ12
15	移植后复发	41	男	2.8	73	46，XY	WT1、KRAS、NF1、EP300、SET-NUP214

**注** ^a^复杂核型89-90，XXXX，4q−，5q−，−6，6q−，+8，+9，+10，11p+×2，−13，−13，−15，−18，+m[5]/46，XX[5]；^b^复杂核型47，XX,7(UPD),LOH(11)(q13q25),LOH(12)(p12p13),del(13)(q13q21),LOH(17)(p11p13),+1；^c^复杂核型46,XY,del（7）（p12p22）[7]/46，iedm，del（11）（q21q25）[1]/46XY,?i(7)(q10)[1]/46,XY[1]

2. 疗效评价：15例患者在疾病不同阶段接受了Ven治疗，均至少完成1个疗程化疗，可评估其疗效，第1疗程用药第21天复查骨髓象。CR率为60.0％（9/15），CRi率为6.7％（1/15）, ORR为66.7％。4例难治患者经Ven联合化疗后3例CR，1例CRi，其中3例接受allo-HSCT；4例难治患者中目前2例无病生存，2例死亡。5例化疗后复发患者3例达CR后行allo-HSCT，目前均无病生存；2例NR死亡。6例移植后复发患者3例达CR，3例NR死亡。3例CR患者中，1例巩固化疗中，2例再次接受allo-HSCT，至随访结束时均存活（表2）。

**表2:** Ven联合多药化疗治疗15例复发难治早期前体T淋巴细胞白血病患者的疗效及转归

例号	方案	疗效	MRD	Ven再诱导CR后移植	≥3级血液不良反应	肺部感染	Ven治疗后复发	生存情况	OS时间 (月)
1	Ven+AZA	CR	2.8×10^−4^	否	有	无	否	死亡	13
2	Ven+AZA	CRi	9.9%	是	有	有	否	死亡	5
3	Ven+DAC	CR	3.5×10^−4^	是	无	无	否	生存	28
4	Ven+Cladribine	CR	2.2×10^−4^	是	有	无	否	生存	19
5	Ven+DAC+HAAG	NR	24.0%	否	有	有	NR	死亡	2
6	Ven+DAC+CAG	NR	26.4%	否	有	有	NR	死亡	0
7	Ven+DAC+HAAG	CR	3.8×10^−5^	是	有	无	是	生存	15
8	Ven+DAC+HAAG	CR	4.8×10^−4^	是	有	有	否	生存	29
9	Ven+DAC+CAG	CR	6.8×10^−4^	是	有	无	否	生存	29
10	Ven+DAC+HAAG	NR	26.8%	否	有	无	NR	死亡	9
11	Ven+AZA	NR	49.8%	否	有	有	NR	死亡	3
12	Ven+AZA	CR	3.6×10^−4^	是	有	无	是	生存	29
13	Ven+AZA	CR	6.2×10^−4^	是	有	无	否	生存	25
14	Ven+DAC	NR	26%	否	有	有	NR	死亡	3
15	Ven+AZA	CR	3.5×10^−4^	否	有	无	否	生存	11

**注** Ven：维奈克拉；AZA：阿扎胞苷；DAC：地西他滨；Cladribine：克拉屈滨；HAAG：高三尖杉酯碱、阿糖胞苷、阿柔比星、G-CSF；CAG：阿克拉霉素、阿糖胞苷、G-CSF；CR：完全缓解；CRi：CR伴血液学不完全恢复；NR：未缓解；MRD：微小残留病；OS：总生存

接受allo-HSCT患者均使用清髓预处理方案（改良BUCY）。15例患者6、12、24个月OS率分别为66.7％、60.0％、52.5％，中位OS时间为17.7个月；全部CR患者12个月DFS率为60.0％，中位DFS时间未达到。10例患者经Ven联合化疗获得CR或CRi后，其中有8例接受了allo-HSCT, 所有移植患者的中位OS时间未达到，未移植患者的中位OS时间为2（0～13）个月，差异具有统计学意义（*P*＝0.009）（图1）。

**图1:**
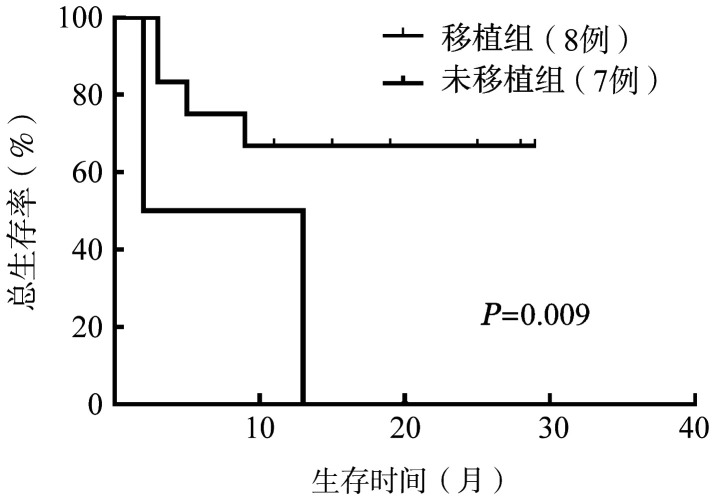
是否接受异基因造血干细胞移植对复发难治早期前体T淋巴细胞白血病患者总生存的影响

3. 不良反应：截至2022年12月30日，15例患者中8例存活，7例死亡，无失访。死亡原因包括疾病进展6例，感染性休克1例。14例患者发生了≥3级血液学不良反应：中性粒细胞减少13例，其中1例在治疗前即存在，12例在治疗期间出现，WBC最低值中位数为0.06（0.01～0.21）×10^9^/L，粒缺中位时间19（18～29）d；贫血11例，其中7例在治疗前即存在，4例在治疗期间出现，中位HGB最低值63（54～112）g/L；血小板减少12例，其中8例在治疗前即存在，4例在治疗期间出现，中位PLT最低值22（11～38）×10^9^/L，无致死性大出血出现。

## 讨论

ETP-ALL中的淋巴母细胞衍生于胸腺中最早期的T细胞前体，在免疫表型和基因水平保留了一些髓系和干细胞特征[7]，现有报道提示该类型起病时原始细胞比例更高、白细胞偏低、诱导化疗反应差、缓解率低、长期生存差[8]。由于其对常规化疗的耐药性导致R/R ETP-ALL的发生率较高，选择合适的再诱导化疗方案使患者获得CR以桥接造血干细胞移植至关重要。

Ven作为单一药物已被证实可改善T-ALL患者的预后，尤其是ETP-ALL，尽管Ven抗肿瘤活性很强，但耐药性的快速出现会限制Ven单药使用[9]。Peirs等[10]和Anderson等[11]报道，Ven可与不同类型的药物联合使用治疗不同亚型的T-ALL，当与阿糖胞苷或地塞米松联合使用时，二者只能在ETP-ALL患者中观察到协同作用。近年来有个案报道提示在R/R ETP-ALL不同治疗线的化疗药物中添加Ven可使患者达到CR，甚至微小残留病（MRD）转阴[12]–[13]。La Starza等[9]报道了3例化疗后复发ETP-ALL患者经Ven联合硼替佐米治疗后获得稳定的细胞学缓解，2例接受HSCT后血液学逐步恢复。Rahmat等[14]报道了1例移植后复发ETP-ALL患者经Ven联合去甲基化药物挽救性治疗后获得细胞及分子学完全缓解。本研究回顾性分析了单中心15例接受Ven联合多药治疗的R/R ETP-ALL患者的疗效以及安全性。1个疗程CR率53.3％，ORR为66.7％。同时我们发现桥接移植患者的OS优于未移植患者，差异有统计学意义。在安全性方面，尽管14例（93.3％）患者发生了≥3级血液学不良反应，但所有患者经治疗后造血功能恢复且无致死性大出血发生；最主要的非血液学不良反应为感染，但仅1例（6.7％）出现严重感染性休克，最终导致死亡；本研究中无患者出现神经系统不良反应，同时无3级及以上脏器不良反应发生。

综上所述，在R/R ETP-ALL不同治疗线的化疗药物中添加Ven疗效值得肯定，治疗相关不良反应可耐受，R/R ETP-ALL患者可选择Ven联合多药化疗方案以期降低肿瘤负荷并获得再次缓解。本研究入组例数较少，且大部分为年轻患者，随访时间较短，确切结论尚需大样本的前瞻性研究进一步证实。今后有必要纳入更多临床病例进行随机对照研究来证实Ven联合多药化疗治疗R/R ETP-ALL患者的疗效和安全性。
